# Expansion, harvest and cryopreservation of human mesenchymal stem cells in a serum‐free microcarrier process

**DOI:** 10.1002/bit.25582

**Published:** 2015-04-20

**Authors:** Thomas R. J. Heathman, Veronica A. M. Glyn, Andrew Picken, Qasim A. Rafiq, Karen Coopman, Alvin W. Nienow, Bo Kara, Christopher J. Hewitt

**Affiliations:** ^1^ Centre for Biological EngineeringLoughborough UniversityLeicestershireLE11 3TUUK; ^2^ Aston Medical Research Institute, School of Life and Health SciencesAston UniversityAston Triangle, BirminghamB4 7ET; ^3^ Centre for Bioprocess EngineeringUniversity of BirminghamB15 2TTUK; ^4^ FUJIFILM Diosynth BiotechnologiesBillinghamTS23 1LHUK

**Keywords:** serum‐free, human mesenchymal stem cell, microcarrier expansion, harvest, cryopreservation, regenerative medicine

## Abstract

Human mesenchymal stem cell (hMSC) therapies are currently progressing through clinical development, driving the need for consistent, and cost effective manufacturing processes to meet the lot‐sizes required for commercial production. The use of animal‐derived serum is common in hMSC culture but has many drawbacks such as limited supply, lot‐to‐lot variability, increased regulatory burden, possibility of pathogen transmission, and reduced scope for process optimization. These constraints may impact the development of a consistent large‐scale process and therefore must be addressed. The aim of this work was therefore to run a pilot study in the systematic development of serum‐free hMSC manufacturing process. Human bone‐marrow derived hMSCs were expanded on fibronectin‐coated, non‐porous plastic microcarriers in 100 mL stirred spinner flasks at a density of 3 × 10^5^ cells.mL^−1^ in serum‐free medium. The hMSCs were successfully harvested by our recently‐developed technique using animal‐free enzymatic cell detachment accompanied by agitation followed by filtration to separate the hMSCs from microcarriers, with a post‐harvest viability of 99.63 ± 0.03%. The hMSCs were found to be in accordance with the ISCT characterization criteria and maintained hMSC outgrowth and colony‐forming potential. The hMSCs were held in suspension post‐harvest to simulate a typical pooling time for a scaled expansion process and cryopreserved in a serum‐free vehicle solution using a controlled‐rate freezing process. Post‐thaw viability was 75.8 ± 1.4% with a similar 3 h attachment efficiency also observed, indicating successful hMSC recovery, and attachment. This approach therefore demonstrates that once an hMSC line and appropriate medium have been selected for production, multiple unit operations can be integrated to generate an animal component‐free hMSC production process from expansion through to cryopreservation. Biotechnol. Bioeng. 2015;112: 1696–1707. © 2015 The Authors. Biotechnology and Bioengineering Published by Wiley Periodicals, Inc.

## Introduction

Regenerative medicine (RM) is a growing field that aims to treat currently unmet clinical indications such as diabetes, cardiovascular disease, and neurological disorders by restoring or maintaining tissue function. Cell‐based therapies form an integral part of the RM industry with the potential, if properly developed, to transform global healthcare. Ever since the term mesenchymal stem cell was first introduced (Caplan, 1991), much anticipation has been generated around the potential for hMSCs to treat and in some cases cure human disease. This anticipation has been largely driven by their relative ease of isolation, their ability to proliferate ex vivo under appropriate culture conditions and their capacity to secrete a range of trophic factors which regulate host immune response and initiate tissue repair (Caplan and Dennis, 2006). Consequently, hMSCs are advancing through clinical development targeting clinical indications such as acute coronary syndrome, stoke, and graft vs. host disease (Heathman et al., 2014).

Despite this progress, many challenges remain before cost effective production, storage, and delivery of hMSCs to the clinic is feasible. For clinical indications where the direct transplant of primary donor hMSCs is insufficient, the expansion of cells in vitro is necessary to increase cell numbers without negatively impacting the therapeutic potency of the cell. The expansion of hMSCs has traditionally taken place in planar tissue culture flasks; however considering that the required manufacturing lot sizes for allogeneic hMSC therapies are likely to be in the order of trillions of cells (Rowley et al., 2012), these systems may not be adequate to fulfill this need. For processes to drive towards the production of cost effective therapies, they should be scalable, compliant with Good Manufacturing Practices (GMP), and be amenable to closed and automated process steps. The addition of microcarriers has been used to culture adherent cells such as hMSCs in suspension (Rafiq et al., 2013) allowing for process scale up, where online monitoring, and control systems can be used to deliver a consistent and cost‐effective hMSC product. Stirred‐suspension bioreactors are currently employed for mammalian cell culture in biopharmaceutical production and therefore their design and operation are well‐understood (Nienow, 2006), with the potential to meet the expected manufacturing demands of large‐scale hMSC therapies.

A key aspect of reducing variation in the process will be reducing and eventually eliminating the use of fetal bovine serum (FBS) from the cell culture medium (Wappler et al., 2013). In addition to lot‐to‐lot variability, there are further process constraints on the use of FBS such as limited supply (Brindley et al., 2012), spiraling cost, potential for pathogen transmission, increased risk of recipient immune reaction (Spees et al., 2004), and reduced scope for process optimization. Furthermore, FBS has been shown to contain immunogenic contaminants which have the potential to negatively impact post‐transplant clinical results (Heiskanen et al., 2007), potentially increasing the regulatory burden placed upon these products. All of these considerations mean that moving towards a serum‐free process would be beneficial in achieving scalable, tunable, and consistent hMSC manufacturing processes. In addition, hMSCs grown in a serum‐free medium have demonstrated increased proliferation rates, up‐regulation of genes important in hMSC function, and down‐regulation of genes involved in the production of proinflammatory cytokines (Crapnell et al., 2013).

Unlike traditional suspension‐based bioprocesses, cell harvesting from the microcarrier surface is critically important as the quality characteristics of hMSCs must be retained throughout this process. Harvesting involves two stages, detachment of hMSCs from microcarriers followed by microcarrier separation from the hMSC product (Nienow et al., 2014). After separation, cell‐based products will undergo a holding time prior to downstream processing and formulation in order to pool the product. Product quality can deteriorate with prolonged holding time (Pal et al., 2008) and should be considered during process development. The large scale manufacture of an allogeneic hMSC product will require long term product storage to decouple production from delivery, in a business model akin to current biopharmaceuticals. Therefore, cryopreservation of hMSCs must be carefully considered to ensure the therapeutic potential of the hMSC product does not deteriorate prior to delivery (Moll et al., 2014).

This study demonstrates for the first time that human hMSCs can be expanded, harvested, separated, cryopreserved, and recovered from a potentially scalable serum‐free microcarrier process. The successful integration of sequential unit operations for a serum‐free hMSC production process from expansion through to cryopreservation provides an important pilot study in the development of a scalable manufacturing process for hMSC therapies.

## Materials & Methods

### Monolayer Culture

Human MSCs were isolated from bone‐marrow aspirate purchased from Lonza (Walkersville, MD, USA) obtained from a healthy donor with informed consent. The local Ethical Committee approved the use of the sample for research. Cells from passage 1 were cryopreserved at a density of 1–2 × 10^6^ cells.ml^−1^ in in a freeze medium containing 90% (v/v) FBS (Hyclone, Belgium) and 10% (v/v) dimethylsulphoxide (Sigma–Aldrich, Gillingham, UK). Cells were grown in T‐flasks seeded at 5,000 cells.cm^−2^ at 37°C in humidified air containing 5% CO_2_. For serum‐based culture, Dulbecco's Modified Eagles Medium (DMEM, 1 g/L glucose; Lonza, UK) supplemented with 10% (v/v) FBS (Hyclone, Belgium), and 2 mM UltraGlutamine (Lonza, Slough, UK) was exchanged every 3 days. For serum‐free culture, the growth surface of T‐flasks was coated with 0.4 μg.cm^−2^ PRIME‐XV^™^ human fibronectin (FN, Irvine Scientific, Santa Ana, CA) and cultured in PRIME‐XV^™^ SFM (Irvine Scientific) as per manufacturer's instructions. On passage, the hMSCs were washed with phosphate buffered saline (PBS) and incubated for 4 min with trypsin (0.25%)/ EDTA (Lonza, UK) or TrypLE Express (Invitrogen, UK). Dissociation reagents were inactivated by the addition of appropriate growth medium and the cell suspension was centrifuged at 220 g for 5 min. The supernatant was discarded and the remaining pellet was re‐suspended in an appropriate volume of culture medium. For serum‐free experiments, hMSCs underwent one adaptation passage in serum‐free medium.

### Spinner Flask Culture

The glass surfaces of 100 mL Spinner flasks (diam. T = 60 mm, BellCo, USA) with a magnetic, horizontal stirrer bar, and a vertical paddle (diam. D = 50 mm) were siliconized with Sigmacoat (Sigma–Aldrich) according to manufacturer instructions. Solid, non‐porous Plastic P‐102L microcarriers of 160‐200 microns (Solohill, USA) were prepared at 500 cm^2^ per 100 mL following manufacturer's instructions. Microcarriers were preconditioned in 50 mL FBS‐containing growth medium for 1 h or coated with 0.1 μg.cm^−2^ FN prior to hMSC inoculation at 6,000 cells.cm^−2^ and cultured in 100 mL of FBS‐containing or PRIME‐XV^™^ SFM at 37°C in humidified air containing 5% CO_2_. A 50% medium exchange was performed every three days for FBS‐containing medium and every 2 days for PRIME‐XV SFM as per manufacturer's instructions. Following inoculation the culture was static for 1 h, after which the culture was agitated constantly at the minimum rate for suspension (N_JS_) found to be 30 rpm, with daily medium samples of 1 mL taken for analysis.

### Analytical Techniques

Analysis of glucose, lactate, ammonia, lactate dehydrogenase (LDH), and total protein was performed using a Cedex Bio‐HT (Roche, Germany). Cell counting, mean cell diameter, and viability (via acridine orange uptake and DAPI exclusion) was performed using a NucleoCounter NC‐3000 automated mammalian cell counter (Chemometec, Denmark). Microcarrier‐based cell counts were obtained whilst the cells were still attached to microcarriers. The following parameters were obtained:

### Specific Growth Rate

$$Specificgrowthrate,\mu =\frac{In(c_{x}(t)/c_{x}(0))}{\Delta t}$$

where μ is the net specific growth rate (h^−1^), C_x_(t), and C_x_(0) are the cell numbers at the end and start of the exponential growth phase, respectively and t is time (h).

### Population Doublings

$$PopulationDoublings,P_{d}=\frac{1}{\log(2)}\cdot \log\left(\frac{c_{x}(t)}{c_{x}(0)}\right)$$

where P_d_ is the number of population doublings, C_x_(t), and C_x_(0) are the cell numbers at the end and start of the exponential growth phase, respectively.

### Specific Metabolite Consumption/Production Rate

$$Specificmetaboliteflux,q_{met}=\left(\frac{\mu }{c_{x}(0)}\right)\cdot \left(\frac{c_{met}(t)-c_{met}(0)}{e^{\mu t}-1}\right)$$

where q_met_ is the net specific metabolite consumption or production rate, μ is the specific growth rate (h^−1^), C_x_(0) is the cell number at the end of the exponential growth phase, C_met_(t), and C_met_(0) are the metabolite concentrations at the end and start of the exponential growth phase, respectively and t is time (h).

### Microcarrier Harvest

The hMSCs were harvested using a recently developed method (Nienow et al., 2014). Briefly, culture medium was removed from the spinner flask and the cells on the microcarriers were washed twice with 100 mL Ca^2+^ and Mg^2+^ free phosphate buffered saline (PBS). The cells were then detached by the use of 50 mL of dissociation enzyme (either TrypLE Express [Invitrogen, UK] or Trypsin [0.25%, w/v]/ EDTA [Lonza, UK]) whilst agitating at 150 rpm for 7–10 min and 250 rpm for the final few seconds. Following detachment, hMSCs were separated from the microcarriers by vacuum filtration using a 60 μm Steriflip^®^ filter (Millipore, USA). The cell suspension was then centrifuged and re‐suspended in the appropriate culture medium.

### hMSC Characterisation

Immunophenotype analysis was performed post microcarrier harvest using an established multiparameter‐based protocol (Chan et al., 2014). Cell viability on microcarriers was assessed by a Nikon Eclipse TS100 fluorescence microscope (Nikon Instruments Europe B.V. UK) using the LIVE/DEAD^®^ (Calcein‐AM/Ethidium Homodimer) Viability/Cytotoxicity Kit (Invitrogen, USA) as per manufacturer's instructions. Colony forming unit‐fibroblast (CFU‐F) efficiency was determined by culturing T‐flasks seeded at 100 cells.cm^−2^ for 14 days, fixing colonies with 4% (v/v) formaldehyde (Sigma–Aldrich), and staining with 1% (w/v) crystal violet solution (Sigma–Aldrich). Colonies with at least 25 cells were counted and CFU‐F potential calculated based on the seeded cell number.

The hMSC differentiation was induced using PRIME‐XV^™^ hMSC Differentiation Medium (Irvine Scientific, USA) as per manufacturer's instructions. After 21 days the differentiation media were removed, cells rinsed with PBS then fixed with 4% (v/v) PFA at room temperature. Adipocytes were stained with 1% (w/v) Oil Red O (Sigma–Aldrich) in isopropanol at room temperature and rinsed with distilled water. Osteoblasts were incubated with 2.5% (v/v) silver nitrate (Sigma–Aldrich) under ultraviolet light (30 min exposure), rinsed with distilled water, and stained with fast violet solution (Sigma–Aldrich) containing 4% (v/v) napthol AS‐MX phosphate alkaline (Sigma–Aldrich) for 45 min at room temperature in the dark. Chondrocytes were stained with 1% (w/v) Alcian blue (Sigma–Aldrich) in 0.1 M hydrochloric acid (Sigma–Aldrich). After 30 min incubation, cells were rinsed three times with 0.1 M HCl. After staining, differentiated cells were visualized under a light microscope (Nikon Eclipse TS‐100, UK).

### Cryopreservation and Thaw

Following detachment and separation from the microcarriers, hMSCs were held in culture medium for 4 h to simulate an expected process pooling time. Harvested cells were then suspended in Prime‐XV^™^ Cryopreservation medium (Irvine Scientific, USA) at 2 × 10^6^ viable cells.ml^−1^. Cells were equilibrated in freezing medium for 30 min at room temperature and aliquots (0.5 ml) were loaded into 1.8 ml cryovials during equilibration. Vials were cooled at 4°C for 5 min then cooled with a Stirling cryocooler (EF600, Asymptote, UK) set to cool at −1°C.min^−1^ to −80°C. Cooled vials were stored under liquid nitrogen vapor for at least one month. Vials were rapidly thawed in a 37°C water bath and cells recovered by growth medium dilution followed by centrifugation. The resultant cell pellet was suspended in growth medium then the cells were seeded into T‐flasks to monitor outgrowth and into fibronectin‐coated multiwell plates to assess cell adhesion.

For the cell adhesion assay, cultured cells were washed with PBS then fixed and permeabilized with BD Cytofix/Cytoperm^™^ kit at 4°C for up to 1 week. After repeated washing, cellular F‐actin was stained with 100 nM Alexa Fluor^®^ Phalloidin with 100 nM 4′,6‐diamidino‐2‐phenylindole counterstain for 30 min in the dark. Stained cells were visualized with a Nikon Eclipse TS100 fluorescence microscope (Nikon Instruments Europe B.V. UK). Cells were harvested during outgrowth and diluted 1:1 with viability stain (10 nM calcein‐AM + 50 μg/ml propidium iodide in PBS) and incubated at 37°C for 7 min in 96 well plates. Stained cells were counted using a Guava EasyCyte 8HT flow cytometer using a standardized analysis protocol with GuavaSoft 2.6 (Merck Millipore, UK).

### Statistical Analysis

Results were deemed to be significant if *P* < 0.05 using a two‐tailed Students *t*‐test.

## Results & Discussion

### Process Map of hMSC Production Process

**Figure 1:**
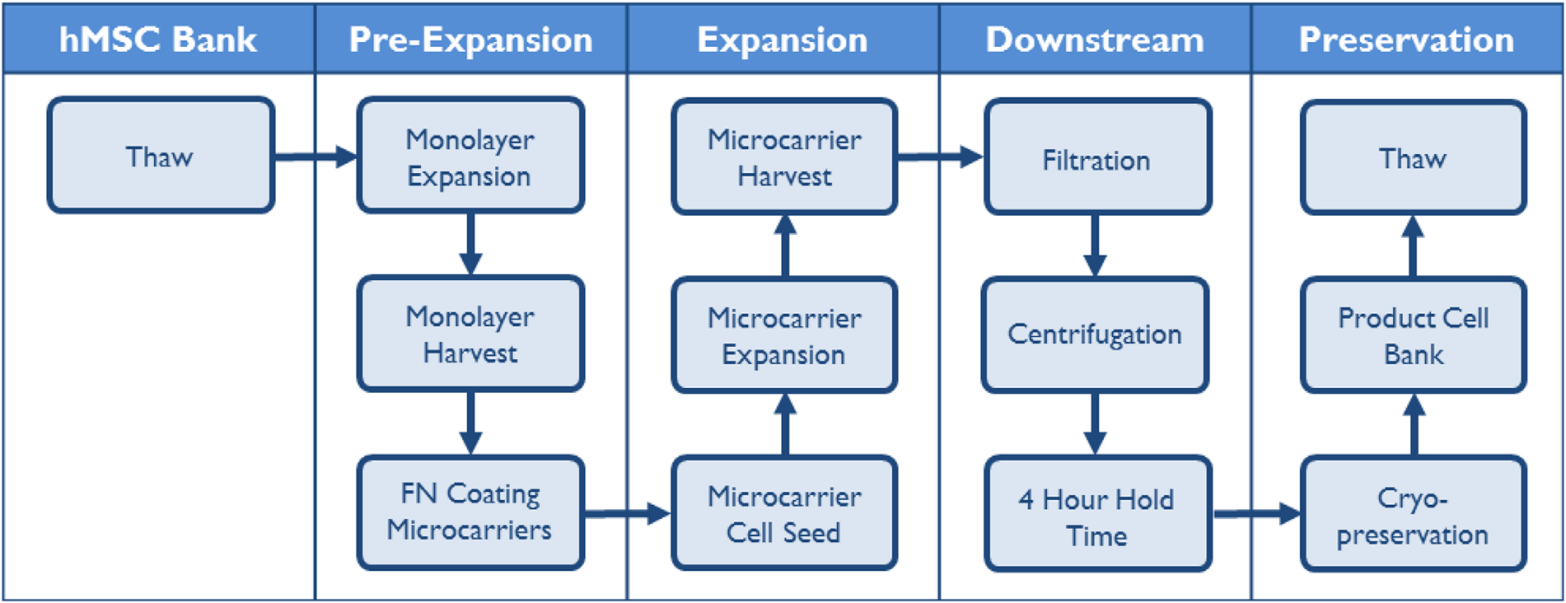
Process map for the serum‐free expansion, harvest, downstream processing and preservation of hMSCs on microcarriers.

Process mapping is a key part of systematic process development and allows for a structured development methodology centered on the concept of integrated unit operations and is being adopted in current biopharmaceutical manufacture (ICH, 2005). Breaking a process down into unit operations allows for the detailed analysis of each process sub‐unit, which can be assessed in terms of its impact on the product characteristics. This flags potential issues or bottlenecks in the process which can then be systematically resolved. Figure 1 shows our simplified process map, which forms the basis for our integrated serum‐free hMSC production process, allowing us to develop individual unit operations to eventually achieve a larger yield of high quality product. The timing of the microcarrier‐fibronectin coating step has been highlighted as a potential bottleneck, which cannot take place immediately prior to the expansion step. A quality risk management approach has highlighted that the quality assurance of the microcarrier coating could not take place in‐process, as a failure event would severely impact subsequent unit operations. This will be addressed during future process development as this unit operation would have to take place in advance to decouple the coating step from the hMSC expansion, reducing the inherent risk in the process.

A further benefit of the process map is that it allows for interchangeability of unit operations that may not be sufficiently scalable for future product requirements. An example of this in our current process is the use of vacuum filtration and centrifugation to separate the hMSCs from the microcarriers, which would be a challenge to operate at large scale. Therefore, during future development, scalable technology would be assessed for the separation, and concentration step such as tangential‐flow filtration. The effect of this unit operation must be assessed in terms of the impact on the product characteristics, to ensure product quality is not compromised. This assessment should be done in a timely manner, as the implications of changing process unit operations late in clinical development can be prohibitive. Therefore by taking this systematic development methodology and utilizing process mapping, potential bottlenecks, and scalability issues can be alleviated at an early stage of development, avoiding costly changes as processes move through clinical development.

### Expansion

As described previously, intensive process scale‐up will be required to meet the clinical and commercial need for these large‐scale allogeneic therapies. For instance, for a typical clinical indication like myocardial infarction, the dose requirements will be in the range of 35–350 million hMSCs per patient (Hare et al., 2009).

**Figure 2:**
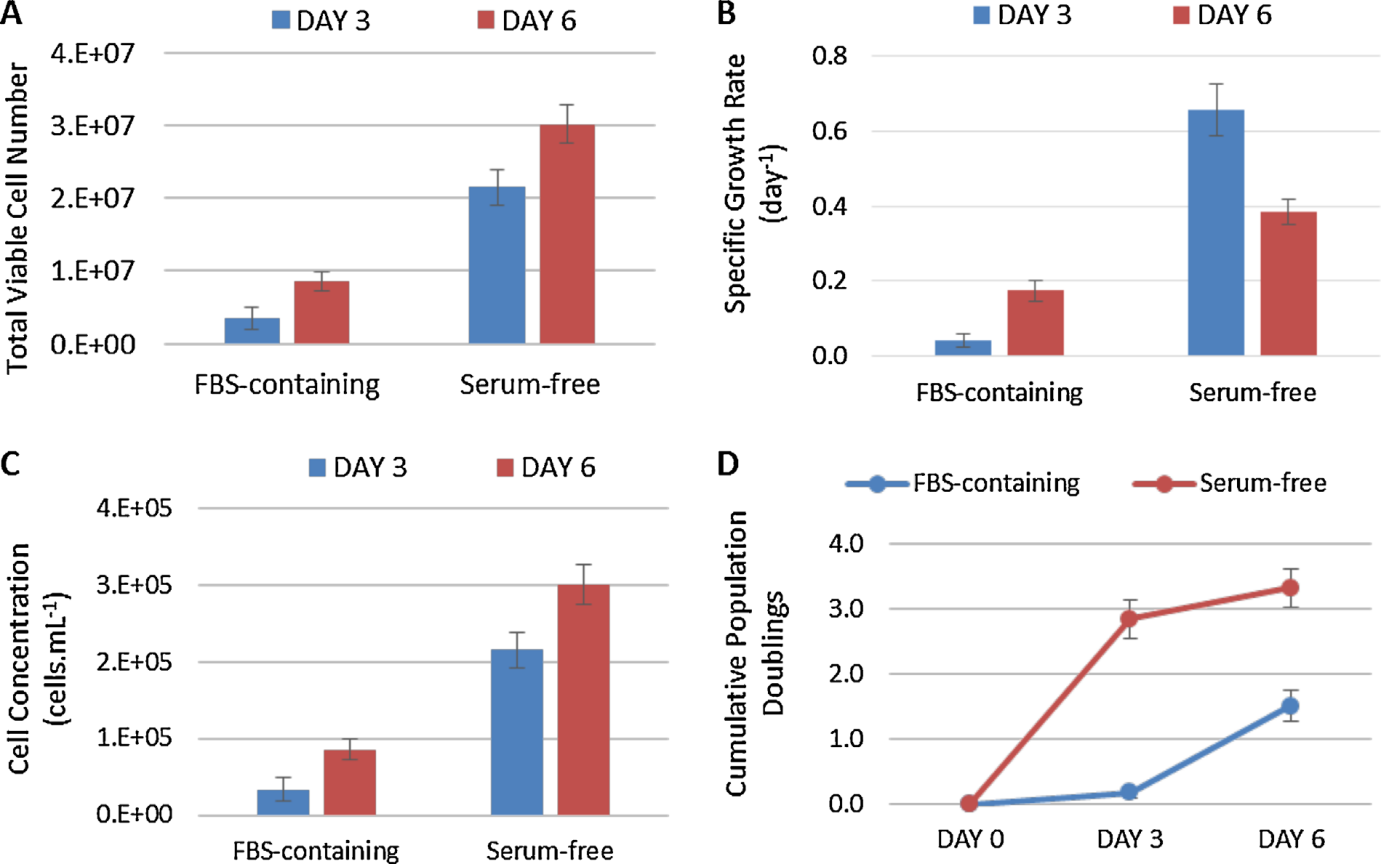
Growth kinetics of hMSCs cultured on microcarriers in FBS‐containing medium and serum‐free medium. Showing (A) Total viable cell number, (B) Specific growth rate, (C) Cell concentration and (D) cumulative population doublings. Data shows mean ± SD n = 3.

The hMSCs were expanded on non‐porous plastic microcarriers in 100 mL spinner flasks over six days in FBS‐containing medium and PRIME‐XV SFM^™^. To facilitate cell attachment without the presence of serum (Hayman et al., 1985), plastic microcarriers were pre‐coated with fibronectin before expansion under serum‐free conditions. For hMSCs expanded in spinner culture with FBS‐containing medium, a final cell density of (8.58 ± 1.37) x 10^4^ cells.mL^−1^ (mean ± SD, n = 3) was reached, corresponding to a fold expansion of 2.86 ± 0.46 (Fig. 2). In contrast, hMSCs expanded in serum‐free medium reached a final cell density of (3.01 ± 0.27) x 10^5^ cells.mL^−1^, corresponding to a fold increase of 10.04 ± 0.88 over the same time period (Fig. 2). Operating under serum‐free conditions gave a 350% increase in hMSC yield, which is significantly higher than serum‐based culture (*P* < 1 · 10^−6^). This difference represents a significant step forward in increasing the lot‐size of hMSC expansion and is comparable to studies which have achieved a cell density of hMSCs cultured on microcarriers of 1–2 × 10^5^ cells.mL^−1^, also under serum‐free conditions (dos Santos et al., 2014; Santos et al., 2011). It was also observed that the hMSC growth kinetics under serum‐free microcarrier culture (0.384 ± 0.014 day^−1^) were significantly higher (*P* < 0.0001) than serum‐free monolayer culture (0.323 ± 0.011 day^−1^). This increase could also be improved further by adding more surface area during the microcarrier culture, reducing the surface area limitation experienced under serum‐free conditions.

**Figure 3:**
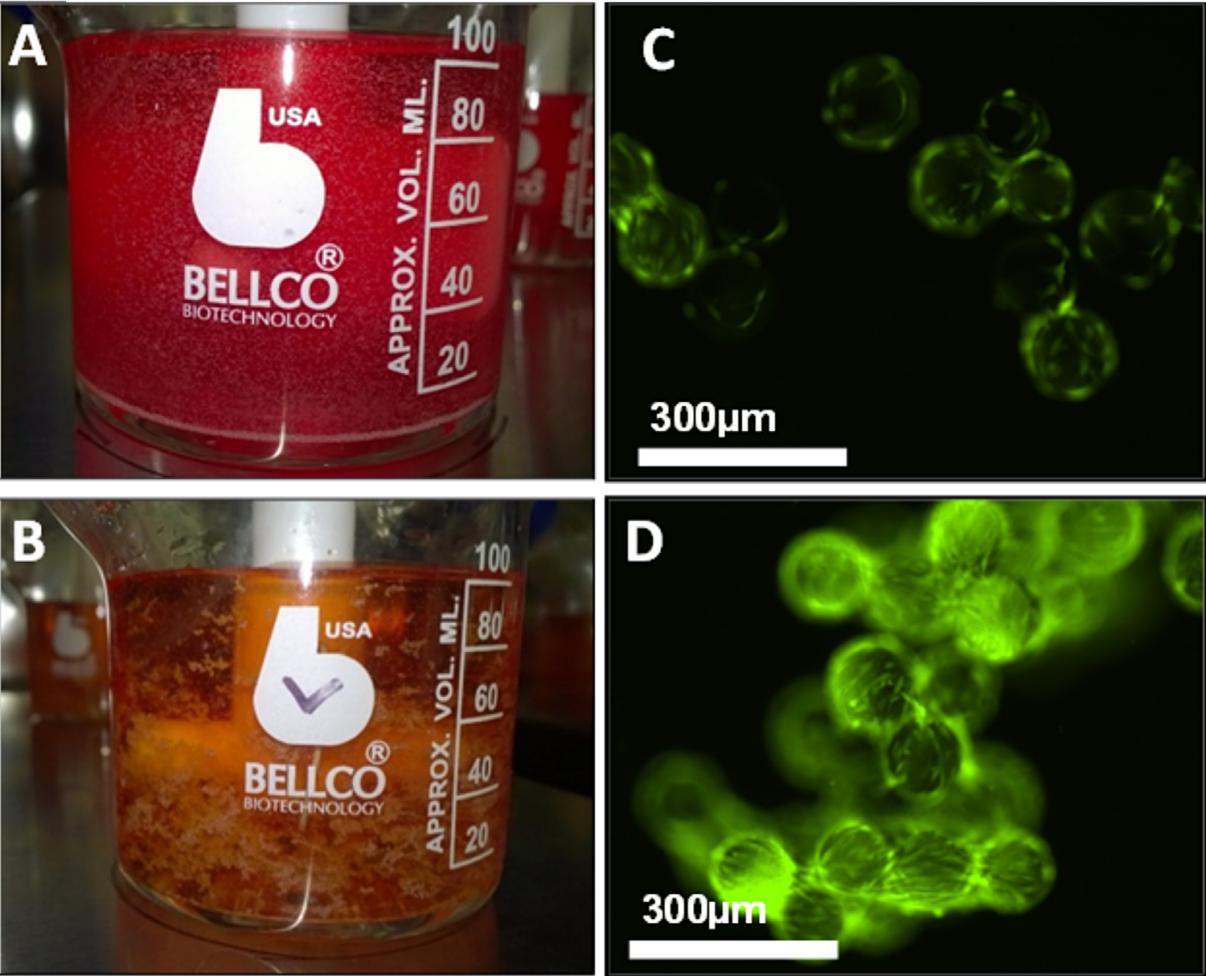
Microcarrier culture of hMSCs on day 5 showing representative images of cell‐microcarrier aggregation. Image of microcarriers in spinner flask with FBS‐containing medium (A) and serum‐free medium (B). Live/dead cell stain of hMSCs on microcarriers in FBS‐containing medium (C) and serum‐free medium (D). Live cells stained with Calcein AM fluorophore (GREEN) and dead cells are stained with ethidium homodimer (RED).

Despite this improvement in hMSC yield under serum‐free conditions, the expansion unit operation is clearly far from optimal. Figure 3 shows the large amount of microcarrier aggregation that occurred in serum‐free culture, which limited the effective surface area available for expansion. The growth kinetics in figure 2B and D, where the hMSC growth between day 3–6 was reduced compared to day 0–3 in serum‐free culture, is also suggestive of surface area limitation. It is likely that microcarrier aggregation is caused by a combination of accelerated cell growth, the microcarriers reaching effective confluence, and the medium sampling process which requires the microcarriers to settle. These potential mechanisms will need to be addressed moving forward as aggregation not only has the potential to reduce cell yield but can also accentuate cell microenvironment heterogeneity (Baraniak et al., 2012) resulting in a cell product of inconsistent quality, although this did not happen here.

**Figure 4:**
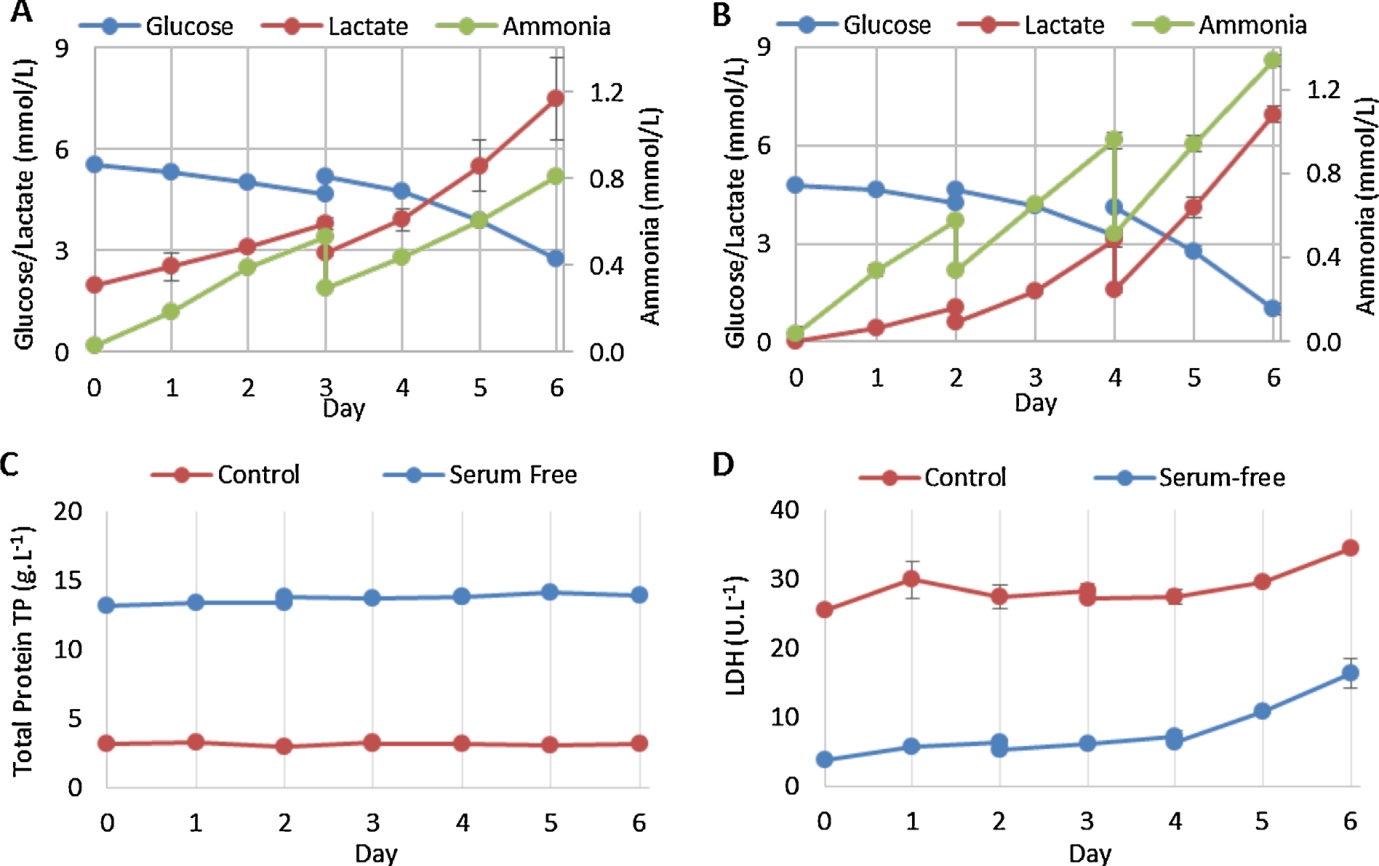
Nutrient and metabolite flux of hMSC expansion on microcarriers. Glucose, lactate and ammonia concentrations in FBS‐containing medium (A) and serum‐free medium (B). Total protein (C) and lactate dehydrogenase concentration (D) are shown for FBS‐containing and serum‐free medium. Data shows mean ± SD, n = 3.

**Figure 5:**
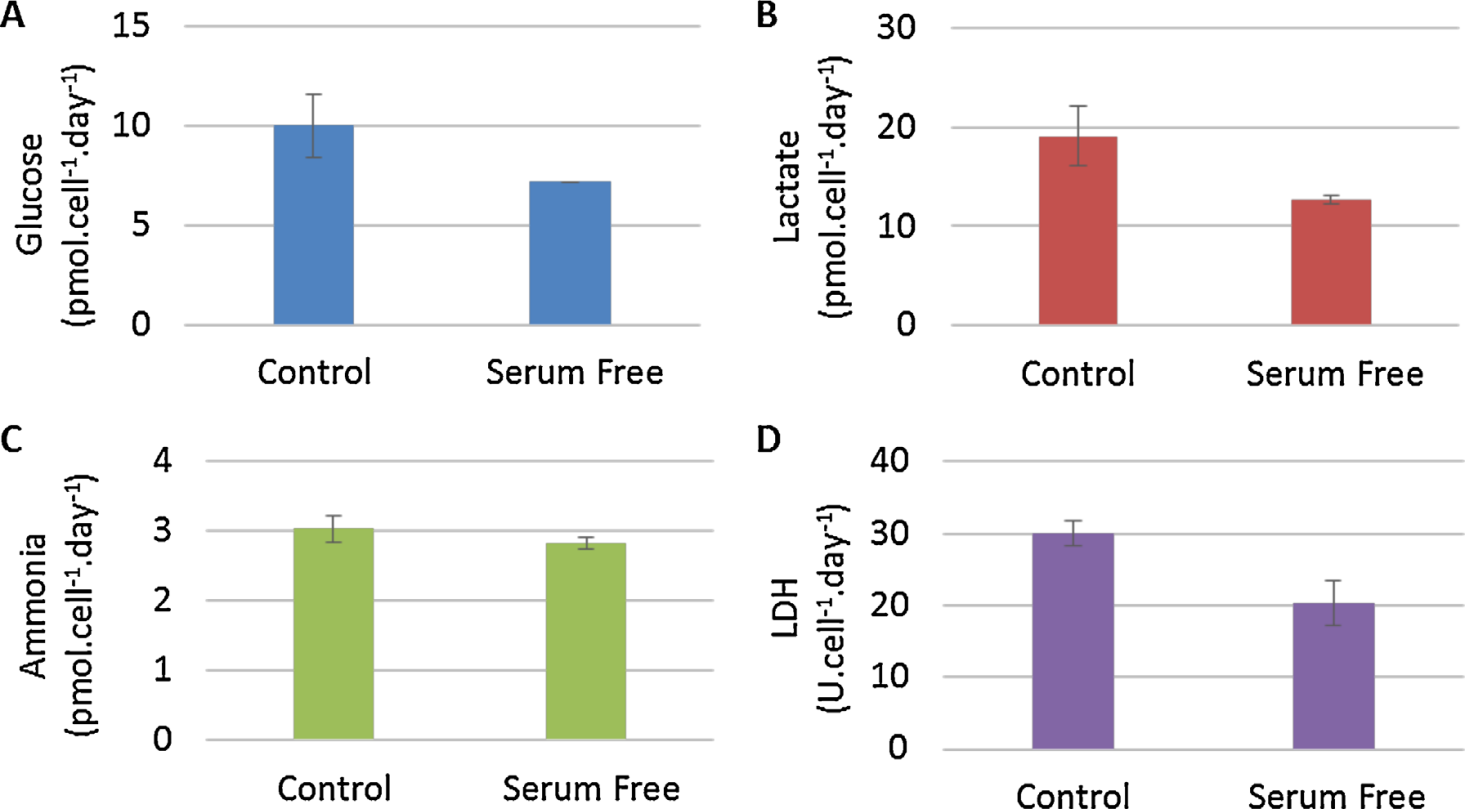
Specific consumption rate per cell of glucose (A) and production rate per cell of lactate (B), ammonia (C) and lactate dehydrogenase (D) for FBS‐containing medium and serum‐free medium. Data shows mean ± SD (n = 3).

**Figure 6:**
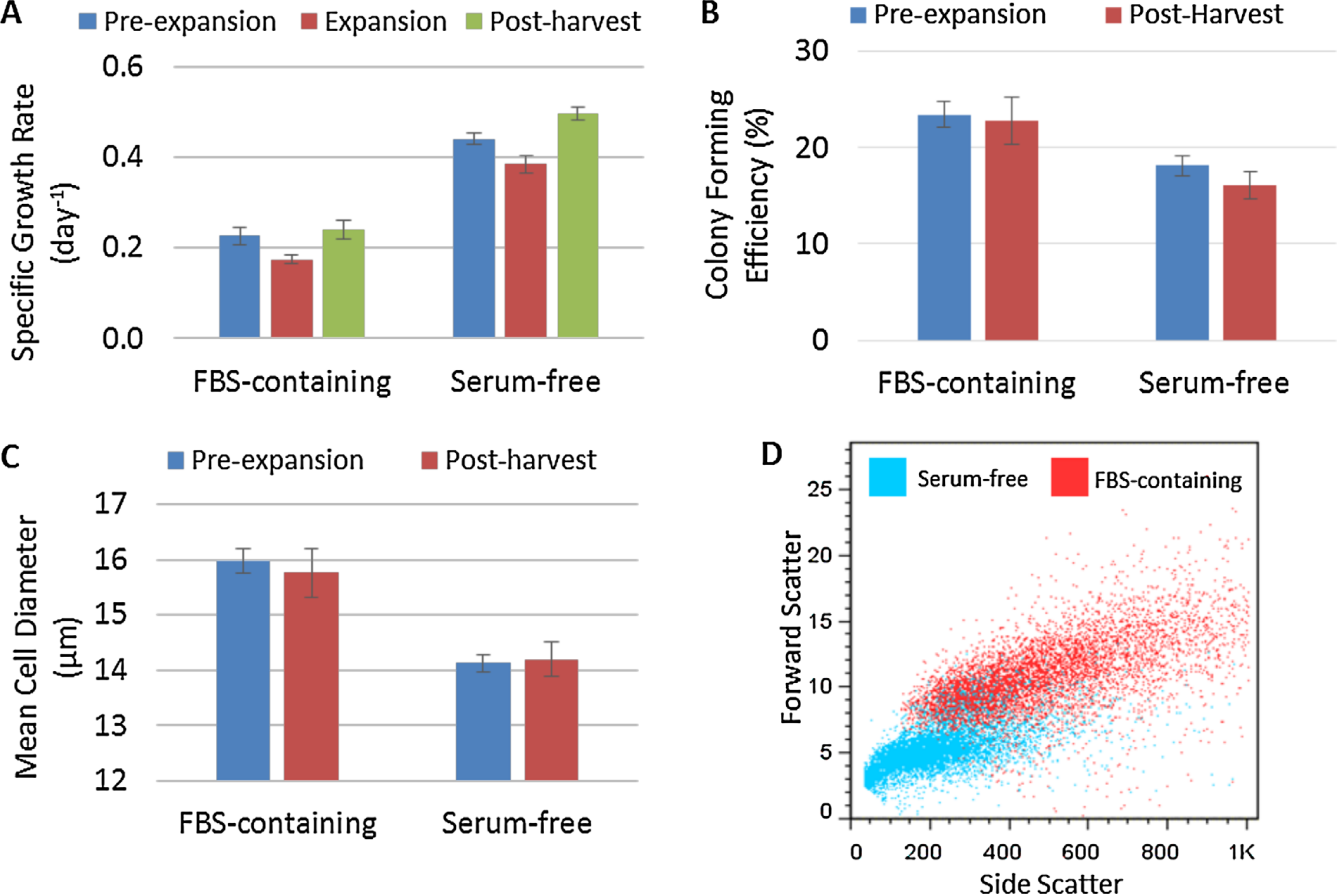
Post‐harvest hMSC quality compared to pre‐expansion demonstrating retention of key attributes, showing (A) specific growth rate, (B) colony forming efficiency, (C) mean cell diameter and (D) forward/side scatter of cell populations confirming difference in mean cell diameter.

Metabolite analysis of microcarrier‐based suspension culture of hMSCs showed differences in the metabolic pathway usage relating to lactate and ammonia production between FBS‐containing and serum‐free cultures. In FBS‐containing medium, hMSCs favored the relative production of lactate over ammonia, whereas the relative production of ammonia over lactate was favored with hMSCs cultured in serum‐free conditions (Fig. 4). Figure 5 shows that the per cell production of lactate was lower in serum‐free culture at 12.63 ± 0.59 pmol.cell^−1^.day^−1^ (mean ± SD, n = 3) compared with 20.81 ± 4.88 pmol.cell^−1^.day^−1^, whereas the production of ammonia was 2.82 ± 0.15 pmol.cell^−1^.day^−1^ in serum‐free, compared to 3.31 ± 0.10 pmol.cell^−1^.day^−1^ in FBS‐containing culture. The estimated yield of lactate from glucose over the entire culture period was 1.91 ± 0.03 and 1.76 ± 0.04 mol_lactate_.mol_glucose_^−1^ for FBS‐containing and serum‐free culture, respectively. We consider the observed differences in these metabolic profiles to be predominantly related to proliferative rate in this instance. The increased proliferative rate coupled with the smaller cell size makes DNA a larger proportion of the total cell biomass under serum‐free conditions (Fig. 6C). Increased ammonia production suggests altered amino acid utilization, which may be related to the increased need for precursors (e.g., glutamine and asparagine) supporting purine, and pyrimidine biosynthesis (Higuera et al., 2012). It is clear that a more detailed metabolic analysis is required and the development of culture medium should consider the impact of these metabolic pathways on cell characteristics. The reduced consumption of glucose and production of lactate per cell under serum‐free conditions does however provide an advantage over serum‐based culture, as the usage, and build‐up of metabolites has the potential to inhibit cell growth as the yield and scale increases.

Growth limiting concentrations of lactate and ammonia for hMSCs, reported as 35.4 mM and 2.4 mM, respectively (Schop et al., 2009), were not reached in any of our microcarrier cultures. Cell death was evaluated by measuring a combination of lactate dehydrogenase (LDH, Lavrentieva et al., 2010) and total protein released. Figure 4C and 4D show these concentrations throughout culture, which indicate only a minimal increase in LDH, and no change in the total protein released. This demonstrates that cell death was minimal throughout the expansion process, despite the increase in microcarrier aggregation, making the fold expansion data a reliable estimate of net proliferative rate.

### Harvest

**Figure 7:**
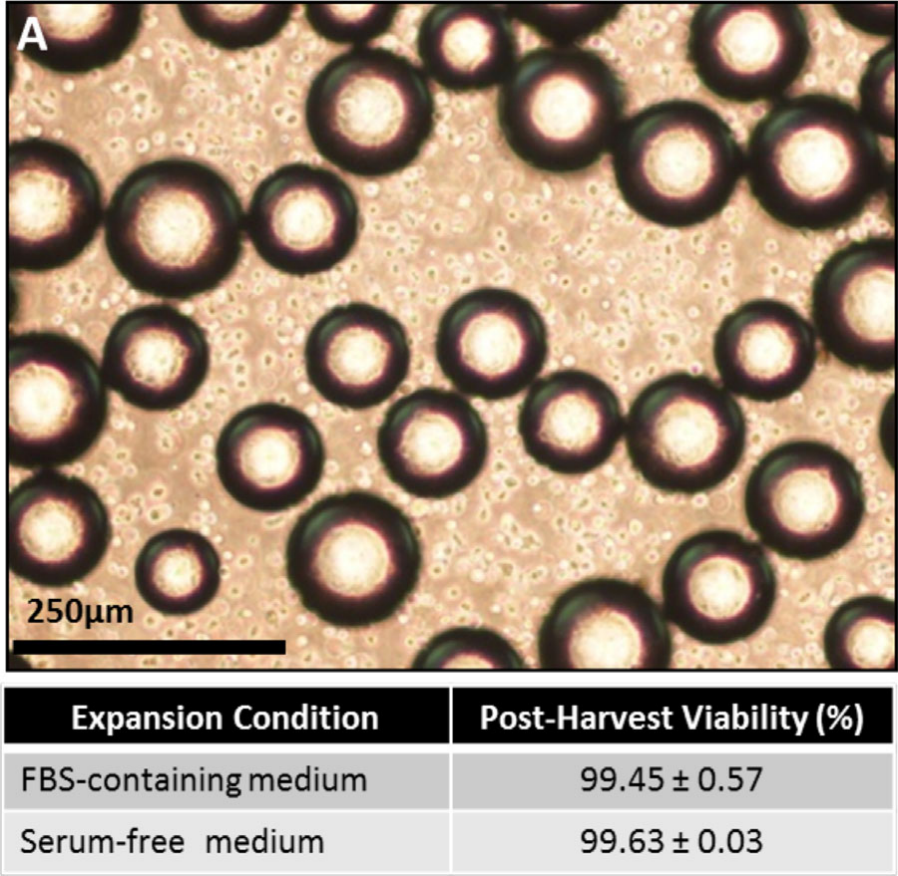
Post‐expansion harvest of hMSCs from microcarriers showing successful hMSC detachment from microcarriers (A). Post‐harvest viability shows high number of intact hMSCs for FBS‐containing medium and serum‐free medium. Data shows mean ± SD, n = 3.

The post‐expansion detachment and separation of the hMSC product from the microcarrier surface, whilst retaining the cell quality, is of critical importance for a scalable production process. The sequential expansion and harvest of hMSCs represents an important step in the successful integration of these unit operations and has been demonstrated previously by our group in a FBS‐based culture (Nienow et al., 2014). The same harvest protocol was modified for this study by replacing trypsin‐EDTA with TrypLE Express for the serum‐free culture to ensure the process was animal‐component free. It was also observed that though microcarrier aggregation and cell number in the serum‐free process was significantly greater than FBS‐based culture, this difference did not limit the effectiveness of the harvest protocol, and the hMSCs were successfully detached from the microcarrier surface (Fig. 7), with post‐harvest cell viability (based on membrane integrity) of 99.63 ± 0.03% (mean ± SD, *n* = 3).

After separating the cells from the microcarriers by vacuum filtration, the hMSCs were held in culture medium at room temperature to simulate a potential large‐scale batch pooling time before centrifugation, freezing medium equilibration, and cryopreservation. This holding step was considered important for a cell‐based product, as a hMSC holding time of greater than 6 h has been shown to negatively impact cellular quality (Pal et al., 2008), which could impose limits on the potential scalability of the bioprocess, depending on the sensitivity of the cell‐based product. This holding process can be broken down into the microcarrier harvest (2 h process) and microcarrier‐cell separation with an ambient hold in culture medium (2 h process). These steps are followed by suspension and equilibration of the cell product in freeze medium for up to 1 h at 4°C prior to cryopreservation, which should be an acceptable exposure time for mixing and dosing thousands of vials or bags with suitable manifold filling systems (Rowley et al., 2012). Tangential‐flow filtration has the potential to wash and concentrate cell products at the large scale within this 2  window (Pattasseril et al., 2013), meaning that the combined harvest, downstream, and preservation timings in this study would still be relevant as the process is scaled‐up further (Heathman et al., 2014).

### Post‐Harvest Characterisation

**Figure 8:**
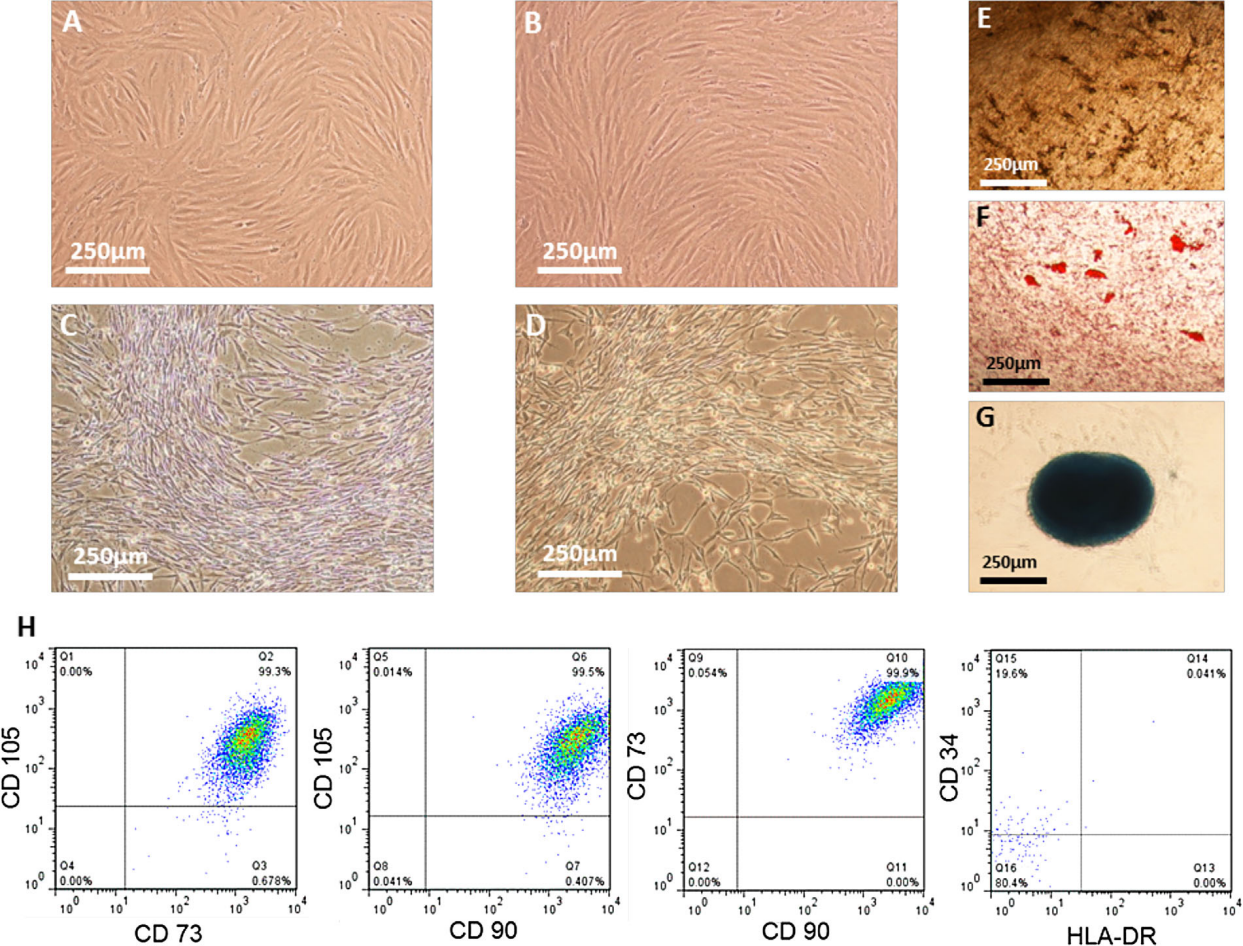
Post‐harvest hMSC characterisation. (A) pre‐expansion and (B) post‐harvest hMSC morphology in FBS‐containing medium. (C) pre‐expansion and (D) post‐harvest hMSC morphology in serum‐free medium. Tri‐lineage differentiation of hMSCs showing (E) osteogenic, (F) adipogenic and (G) chondrogenic potential post‐harvest in serum‐free medium. Multiparameter flow cytometry showing dual gating of CD73, 90, 105, 34 and HLA‐DR for hMSCs post‐harvest from serum‐free microcarrier culture (H).

To ensure that the microcarrier‐based expansion and harvest unit operations have not had a detrimental effect on identity and quality, hMSC characteristics have been evaluated immediately post‐harvest. The primary objective for this is to demonstrate that the hMSCs conform to the International Society for Cellular Therapy (ISCT) criteria (Dominici et al., 2006). Figure 8 shows the compliance of the hMSCs with the ISCT criteria, by adherence to tissue culture plastic, demonstrating the same morphology post‐harvest as was demonstrated pre‐expansion, and differentiation down the osteogenic (Fig. 8E), adipogenic (Fig. 8F), and chondrogenic (Fig. 8G) lineages. The post‐harvest hMSC immunophenotype can be seen in Figure 8H which shows the co‐expression of positive markers CD73, 90, and 105 at greater than 99% as well as the expression of HLA‐DR at less than 2% (Chan et al., 2014). There was an increase in the positive expression of CD34 above the 2% positive threshold, which can be attributed to an increase in non‐specific antibody binding caused by the culture of hMSCs on a fibronectin substrate (data not shown), and has previously been reported to be positive for adipose derived hMSCs (Wagner et al., 2005).

The successful development of a hMSC manufacturing process relies on the characterization of product identity and quality at each unit operation (Carmen et al., 2012). The development of clinical indication specific hMSC quality assays has proved to be complex, owing to their unique and multifactorial putative mechanism of action. Without definitive quality assays, we rely on surrogate assays to measure cell attributes that are known to be related to aspects of hMSC quality. Figure 8A shows the outgrowth of hMSCs before, during and after expansion, with no decline in the proliferative potential observed across these unit operations. This suggests that hMSCs have not experienced detrimental conditions during the microcarrier expansion and harvest process that could have affected their proliferation potential (Sethe et al., 2006).

Colony forming potential has been highlighted as an important assay for the quality of hMSC preparations (Pochampally 2008) and is known to deteriorate during culture (Schellenberg et al., 2012). Figure 8B shows the maintenance of colony forming potential from pre‐expansion to post harvest for both conditions, which further demonstrates that the hMSCs have not been damaged during the harvest, and separation process.

The size of hMSCs in culture is known to increase as they undergo cellular senescence (Wuchter et al., 2015), and should therefore be tracked throughout expansion and harvest to ensure it remains stable. Figure 8C demonstrates that the mean cell diameter remained stable throughout culture, with significantly smaller hMSCs produced under serum‐free culture (*P* < 0.05). Without the availability of a robust potency assay to determine the implications of a smaller cell size in serum‐free culture, it is not known how this will affect in vivo hMSC quality attributes. Despite this, clinical work has demonstrated that smaller hMSCs reduce the potential for vascular obstructions and stroke following the intra‐artery injection of cells (Ge et al., 2014), as well as reducing capillary entrapment (Dreher et al., 2013). These observations suggest that a smaller cell size may not only be beneficial in terms of obtaining a higher number of cells per area for expansion, but might also be advantageous in product delivery. This possibility raises a question of whether the therapeutic potential of the cell is related to size and whether we need to think not only in terms of cell number but also in terms of product biomass for production and delivery of cell therapies. Cellular enlargement has been associated with the development of professional secretory cells such as plasma cells (Shaffer et al., 2004), with more organelles and increased protein synthesis. Considering that protein secretion is a putative mechanism of action of hMSCs in vivo, the relation of cell size to secretory capability of hMSCs should be clinically evaluated post‐delivery.

### Cryopreservation and Recovery

**Figure 9:**
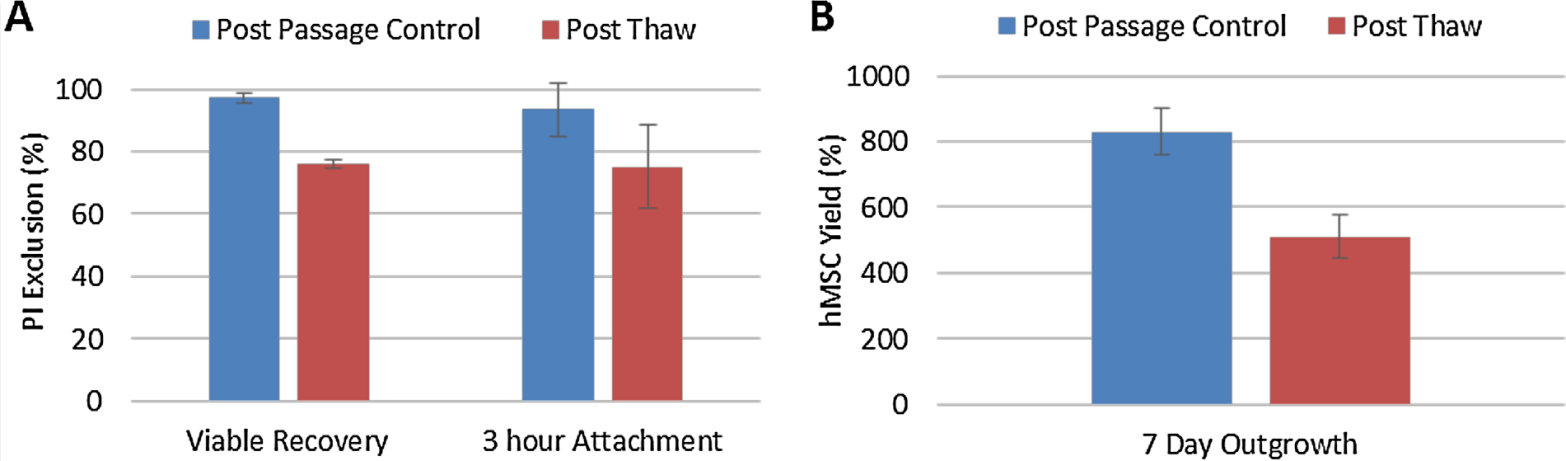
Post‐thaw hMSC recovery following serum‐free cryopreservation, showing (A) post‐thaw recovery and 3 h cell attachment based on PI exclusion. Post‐thaw hMSC outgrowth (B) following serum‐free cryopreservation. Data shows mean ± SD (n = 3).

The hMSCs harvested from serum‐free spinner cultures were preserved using a serum‐free freezing medium and a slow‐freezing cryopreservation process. After thawing, cell viability (by membrane integrity) decreased to 75.8 ± 1.4% as a consequence of the cryopreservation process (Fig. 9A). However, this value remains above the FDA guideline for cell‐based therapies of 70% (FDA, 2008). A similar number of cells were recovered after 3 h in culture, based on their sustained adherence to fibronectin without loss of membrane integrity (Fig. 9A). It is important to note that this post‐thaw recovery is comparable to studies where hMSCs have been immediately processed from monolayer culture (Liu et al., 2010), without the microcarrier harvest, filtration, and holding time steps.

**Figure 10:**
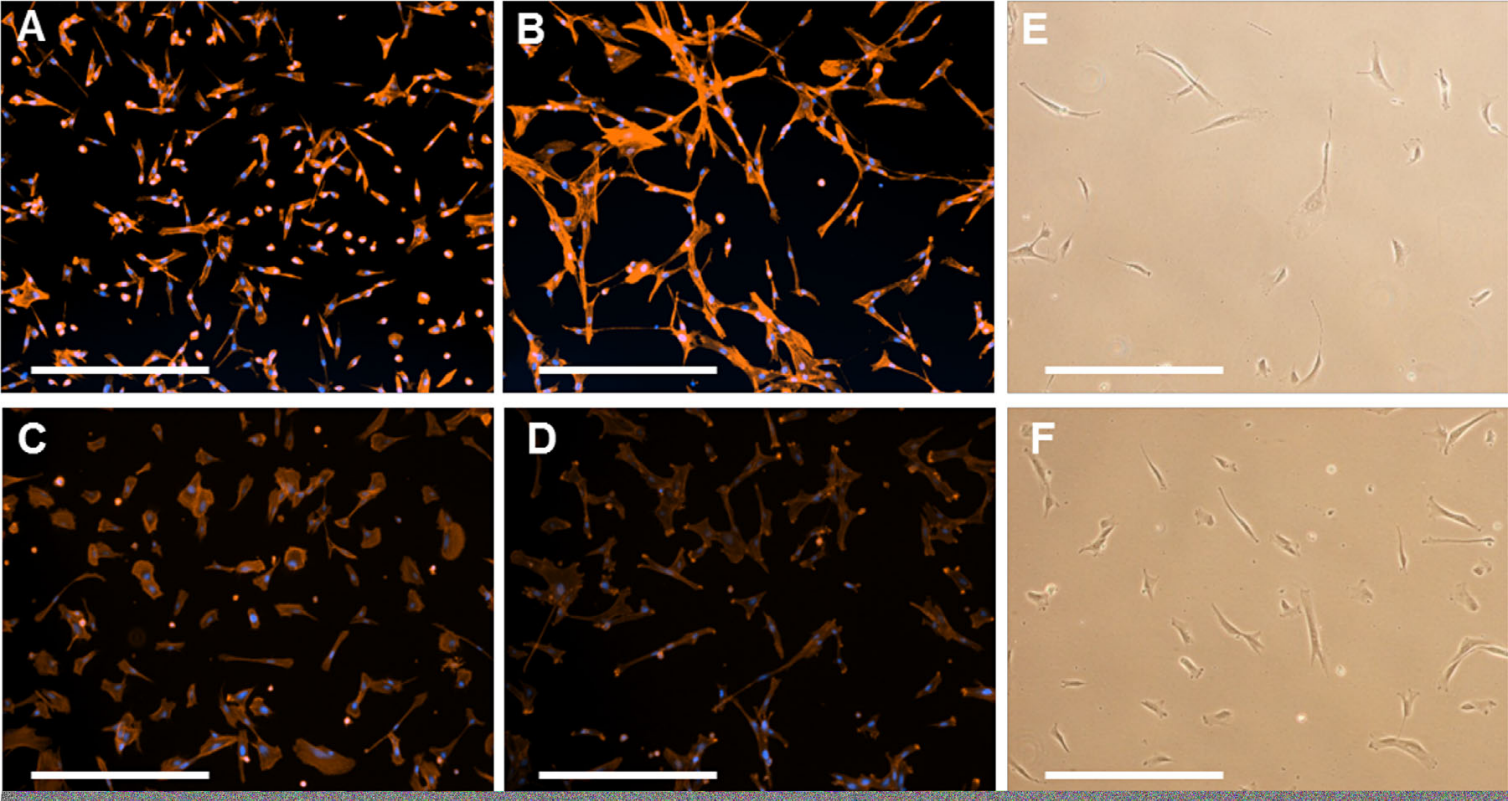
Post‐thaw hMSC recovery following serum‐free cryopreservation, demonstrating formation of F‐Actin cytoskeleton (A) 3 h, (B) 24 hours post thaw (C) 3 h post‐passage control and (D) 24 hours post‐passage control. Phase contrast images show day 2 hMSC morphology post‐thaw (E) and post passage control (F). Scale bar = 250 μm.

Despite the initial cell loss post‐thaw, a 500% increase in cell yield was obtained after 7 days in monolayer culture (compared with 800% for unpreserved post‐passage control), showing that recovered cells were able to proliferate normally (Fig. 9B). Recovered cells also displayed comparable morphology to unpreserved cells after 3 h and 24 h of culture on fibronectin, with signs of matured cell‐matrix interactions, cell elongation (indicative of motility, Huttenlocher and Horwitz, 2011), and recovery of F‐actin networks (Fig. 10). These observations demonstrate that hMSCs can be cryopreserved and recovered from a microcarrier expansion, harvest, and holding process, with comparable cell yields to traditional monolayer harvest and immediate cell preservation.

## Conclusions

This study has demonstrated the feasibility of a serum‐free microcarrier process for the expansion, harvest and preservation of hMSCs. The integration of multiple process unit operations is an important step in developing a microcarrier‐based expansion process capable of meeting the lot sizes required for clinical applications. The hMSC identity and quality have been maintained throughout every unit operation of this integrated process, culminating with the successful recovery of hMSCs from the cryopreservation step. Mapping has provided a robust process understanding from end‐to‐end, which can be broken down into individual unit operations and optimized for hMSC yield, quality, and consistency. The systematic development of a process control system for the expansion step will form a key part of this, as well as the identification and mitigation of bottlenecks to further streamline the process.

This study has been funded by the Engineering and Physical Sciences Research Council (EPSRC), the Biotechnology and Biological Sciences Research Council (BBSRC) and FUJIFILM Diosynth Biotechnologies.
